# The cilium in lights: new views of an ancient organelle

**DOI:** 10.1186/1741-7007-11-74

**Published:** 2013-07-03

**Authors:** Iain A Drummond

**Affiliations:** 1 Nephrology Division, Massachusetts General Hospital, 149 13th Street, Rm 149-8000, Charlestown, MA 02129, USA

## Abstract

Nearly all cells have cilia, microtubule based projections from the apical cell surface, and nearly all cells use them in essential and unique ways. How mammalian cilia function *in vivo* as mechanosensors, signal transducers, or in fluid propulsion has been difficult to study due to lack of tools for live imaging of cilia. A new transgenic mouse that enables tissue-specific GFP labeling of cilia creates new opportunities for using live imaging to understand cilium function and to better characterize the many genetic models of ciliopathies.

See research article: http://www.ciliajournal.com/content/2/1/8

## The cilium at the crossroads of development and disease

The cilium is a multifunctional organelle now known to be a focal point of cell signaling as well as an essential mechanical device that cells use to propel themselves or surrounding fluid. Ciliary dysfunction in ‘ciliopathies’ leads to a wide range of human disease pathologies [1]. The confluence of interest in the cilium over the last decade comes from investigation in fields ranging from vision to obesity and from biological problems as diverse as hedgehog signaling in the neural tube and mucociliary transport in the trachea. Cilia form the backbone of the photoreceptor light-harvesting outer segments [2] and function in hypothalamic neurons to mediate satiety signals [3]. Sensory, non-motile cilia are required for Gli processing in the hedgehog signaling cascade [4] while motile cilia beat in a polarized fashion to move fluid and mucus [5]. In the kidney, many ciliopathy mutations lead to kidney cyst formation, an outcome thought to be due to a failure in the normal mechanosensation of fluid flow in renal tubules [1]. Despite decades of work, many questions remain about how cilia function *in vivo*. For instance, in the kidney, conditional knockout of genes essential for ciliogenesis in adult animals does not result in immediate or uniform kidney cyst formation, raising questions of how cilia detect fluid movement *in vivo* and how they function in normal epithelial homeostasis [3]. Much has been learned about tissue-specific functions of ciliary proteins from the tissue pathology associated with genetic models of ciliopathies while vertebrate cell culture models have until now provided better access to live imaging of cilia. For instance, most of what we know about ciliary mechanosensation in kidney epithelia is derived from studies of the effect of artificial fluid flow on ciliated epithelial cells in culture [6]. Imaging mammalian cilia *in vivo* has largely relied on DIC optics to detect motile cilia in a limited number of organ contexts where cilia can be made accessible, for instance in the isolated embryonic node [7] or the tracheal surface epithelium [8]. The recent introduction of innovative transgenics that specifically label live cilia *in vivo* with green fluorescent protein (GFP) promises to open up new opportunities to understand the function of cilia in their normal organ context.

## Watching cilia work

Live, *in vivo* imaging of cilia using fluorescent protein reporter transgenes has been a mainstay of studies using *Caenorhabditis elegans* for examination of ciliogenesis and ciliary sensory functions. Transgenic GFP fusion proteins have been used to report *in vivo* rates of ciliary transport (intraflagellar transport; IFT) [9], the delivery of ciliary membrane proteins, and localization of ciliary proteins to the transition zone, a ‘gatekeeper’ of ciliary protein content [9]. More recently, in the zebrafish, transgenic GFP-labeled cilia have revealed requirements for planar polarity proteins in ciliary polarization [10] and permitted precise measurements of *in vivo* ciliary motility [11]. A key advance provided by GFP labeled cilia is the ability to distinguish motile from non-motile cilia *in vivo*. For instance, in the embryonic mouse node, two populations of cilia are proposed to co-exist and be essential for the determination of embryonic left-right asymmetry. Motile cilia drive fluid in a leftward direction while non-motile, mechanosensory cilia are proposed to detect this fluid flow and initiate asymmetric signaling on the left side of the embryo [12]. While it may seem obvious, in order to distinguish motile cilia from non-motile sensory cilia it is essential to examine living tissue. With DIC optics it has been possible to image motile cilia; however, unlabeled sensory, non-motile cilia can be very difficult to detect reliably against a background of other cells and their deflection by fluid movement has not been well studied. Advances in the imaging of cilia using transient *ex vivo* lentiviral infection of mouse embryos with GFP targeted to cilia have been reported [13], but this approach is inherently variable and somewhat labor intensive. A new transgenic Cilia^GFP^ mouse harboring a floxed, cilia-targeted GFP transgene integrated in the ROSA locus promises to become the new standard for *in vivo* visualization of cilia. In their recent report, O’Connor *et al*. [14] describe the Cilia^GFP^ mouse and show that cilia can be labeled *in vivo* with a transgene-encoded somatostatin receptor 3-GFP fusion protein (Sstr3::GFP) using ubiquitous and cell-type-specific Cre lines. Live imaging of kidney tubules reveals that fluid flow in the mouse nephron is sufficient to force the cilia to lie flat against the cell surface in the direction of flow, while at reduced heart rate cilia show a pulsatile and coordinated back and forth motion. After years of debate and supposition about how sensory cilia behave *in vivo* and how they may be linked to disease, the work from O'Connor *et al*. finally provides empirical measurements of ciliary deflection and movement that will be essential for deducing their *in vivo* function. How is ciliary deflection transduced as a cellular signal *in vivo*? Do cilia have a characteristic bending pattern and is this important in mechanotransduction? Or do some kidney cilia move on their own using internal motors (dyneins)? These questions and many others are now within the grasp of researchers. The Cilia^GFP^ mouse will also be useful for studies of the brain where primary cilia in the hippocampus as well as motile cilia on ependymal cells of the ventricles have been shown to be well labeled. The Cilia^GFP^ mouse approach will impact studies of how cerebrospinal fluid movements may affect brain development [15].

## Ciliary inputs and outputs

Finally, it is clear that cilia are not static structures but respond to their environment with not only movement but also with changes in structure or length. Are cilia an element in homeostatic responses to tissue injury? Do they undergo dynamic length changes under stress? The approach to answering these questions is now in hand. The Cilia^GFP^ mouse suggests new approaches, integrating observations on the behavior of cilia with *in vivo* measurements of cell signaling. Imaging cilia in the Cilia^GFP^ mouse coupled with genetically encoded biosensor transgenics (for instance, GCaMP3 for simultaneous measurements of intracellular calcium [16]) promise to bring highly relevant, *in vivo* physiology assays to genetic models of ciliopathy and reveal new and exciting details of ciliary function.
